# Decoding the lncRNA-miRNA-mRNA network in sepsis-induced lung injury: from pathogenesis to extracellular vesicle-based therapy

**DOI:** 10.3389/fimmu.2026.1701440

**Published:** 2026-01-28

**Authors:** Yating Wei, Weiye Gong, Yuhua Wei, Xiaohong Jiang, Chaoqian Li, Rongzong Ye

**Affiliations:** 1 Department of Emergency Medicine, The First Affiliated Hospital of Guangxi Medical University, Nanning, China; 2 Department of Pulmonary and Critical Care Medicine, the First Affiliated Hospital of Guangxi Medical University, Nanning, China; 3 Department of Pediatrics, The People’s Hospital of Guangxi Zhuang Autonomous Region, Nanning, China; 4 Department of Geriatric Respiratory Medicine, The First Affiliated Hospital of Guangxi Medical University, Nanning, China; 5 Department of Critical Care Medicine, The First Affiliated Hospital of Xiamen University, School of Medicine, Xiamen University, Xiamen, China

**Keywords:** cell-specific regulation, competing endogenous RNA, extracellular vesicles, lncRNA-miRNA-mRNA axis, sepsis-associated acute lung injury

## Abstract

Sepsis-induced acute lung injury (S-ALI) represents a life-threatening condition with complex molecular pathophysiology and limited therapeutic options. Emerging evidence highlights the critical role of competing endogenous RNA (ceRNA) networks, particularly long non-coding RNA (lncRNA)–microRNA (miRNA)–mRNA axes, in orchestrating cell type-specific responses during S-ALI. This review synthesizes recent advances illustrating how these regulatory circuits modulate alveolar epithelial apoptosis, endothelial permeability, macrophage polarization, and neutrophil infiltration, thereby driving inflammation, barrier dysfunction, and immune dysregulation. Furthermore, we explore the promising therapeutic potential of engineered extracellular vesicles for targeted delivery of ceRNA components—such as miRNA mimics or lncRNA inhibitors—to precisely manipulate these networks. Despite progress, significant challenges remain, including model translatability, functional redundancy, and delivery efficiency. Overcoming these hurdles may unlock novel strategies for treating S-ALI, moving toward personalized and context-specific interventions.

## 1 Introduction

Sepsis is a life-threatening condition characterized by organ dysfunction resulting from a dysregulated host response to infection, accompanied by systemic inflammation and immune imbalance (1). According to the 2017 Global Burden of Disease Study, sepsis affects approximately 48.9 million individuals annually and is responsible for 11 million deaths worldwide, accounting for 19.7% of global mortality (2, 3). Due to its substantial morbidity and mortality, sepsis remains a critical public health challenge. A frequent and early complication among septic patients is acute lung injury (ALI), which exhibits a high incidence and can progress to acute respiratory distress syndrome (ARDS). Sepsis-associated ARDS carries a mortality rate as high as 70–90% (4, 5). ARDS/ALI is defined by rapid-onset respiratory failure, non-cardiogenic pulmonary edema, severe hypoxemia, and dyspnea (6). The underlying pathogenesis involves inflammatory dysregulation and disruption of the alveolar barrier, driven by mechanisms such as increased endothelial and epithelial permeability, cytokine storm, hyperactivation of immune responses, and coagulation dysfunction (7, 8). Despite being the most common cause of ARDS, the molecular mechanisms underlying sepsis-induced acute lung injury (S-ALI) are not yet fully elucidated.

MicroRNAs (miRNAs) are endogenous small non-coding RNAs, approximately 22 nucleotides in length, that post-transcriptionally regulate gene expression by binding to complementary sequences within the 3′-untranslated regions of target mRNAs, leading to mRNA degradation or translational repression (9, 10). Long non-coding RNAs (lncRNAs), defined as transcripts exceeding 200 nucleotides with limited protein-coding potential, represent another important class of regulatory molecules (11). A widely recognized mechanism of lncRNA function involves their role as competitive endogenous RNAs (ceRNAs), acting as molecular sponges that sequester miRNAs through shared binding sites, thereby modulating the availability of miRNAs for their target mRNAs. Although the ceRNA hypothesis has been subject to some debate, it remains a dominant framework for understanding lncRNA-mediated regulatory networks (12–14). Additionally, certain lncRNAs serve as primary transcripts for miRNA biogenesis (15).

**Figure 1:**
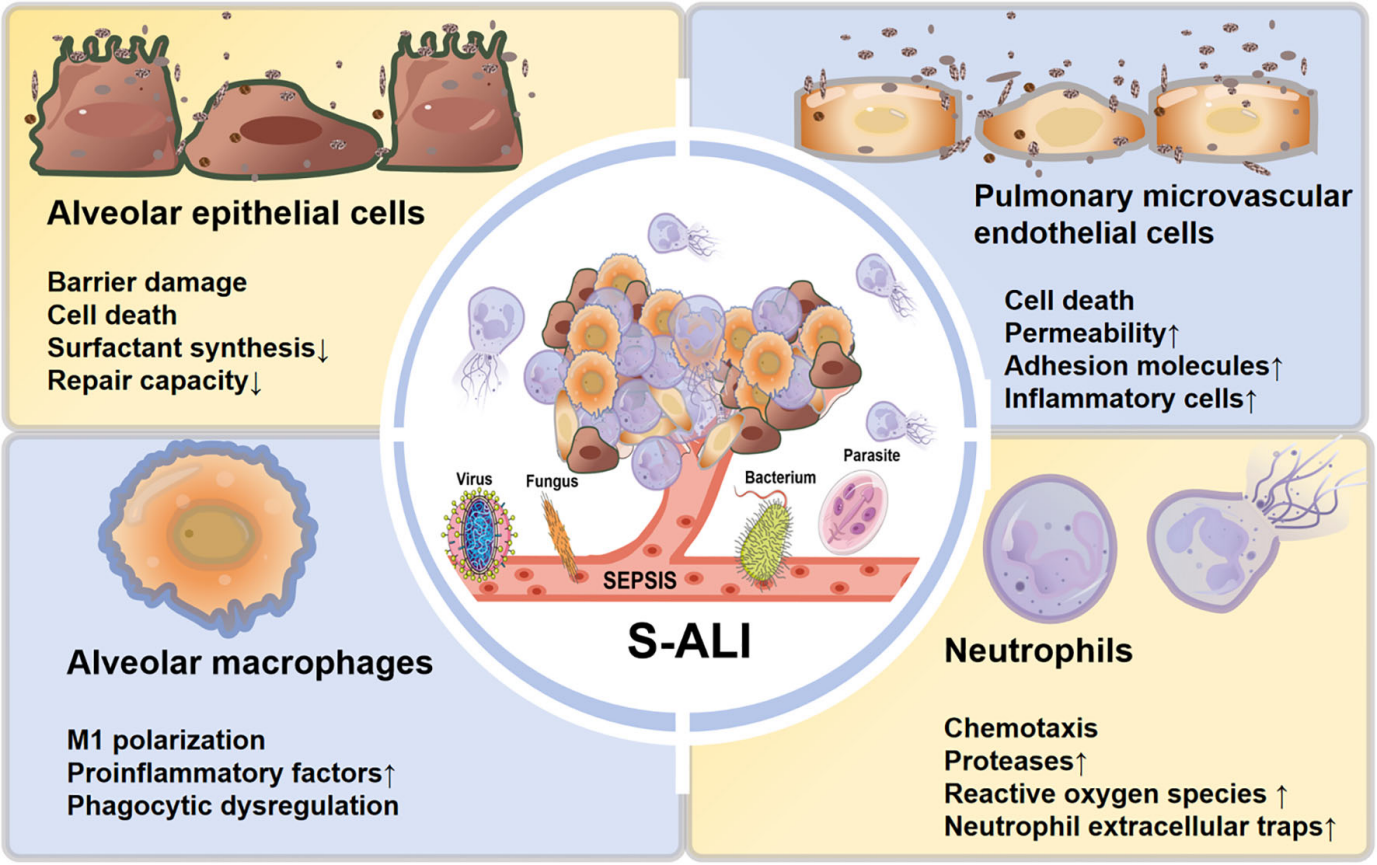
Schematic illustration of sepsis-induced acute lung injury: systemic inflammation and its impact on key pulmonary cell dysfunction. Sepsis, initiated by pathogens including viruses, bacteria, fungi, and parasites, triggers a systemic inflammatory response that culminates in S-ALI. This process is mediated through the dysfunction of core pulmonary cell populations: alveolar epithelial cells suffer barrier damage, cell death, reduced surfactant synthesis, and impaired repair capacity; pulmonary microvascular endothelial cells exhibit increased permeability and upregulation of adhesion molecules, promoting inflammatory cell infiltration; alveolar macrophages undergo M1 proinflammatory polarization, leading to excessive release of proinflammatory factors and phagocytic dysregulation. Subsequently, recruited neutrophils exacerbate tissue damage through enhanced chemotaxis, release of proteases and reactive oxygen species, and formation of neutrophil extracellular traps (NETs). The concerted dysfunction of these cells drives the pathogenesis of sepsis-induced acute lung injury.

Growing evidence highlights the significance of lncRNAs and miRNAs in regulating pathophysiological processes during S-ALI. For instance, lncRNA gadd7 has been shown to mediate mitophagy and promote apoptosis in type II alveolar epithelial cells (AEC II) (16). Similarly, elevated levels of miR-223-3p in plasma-derived extracellular vesicles (EVs) from septic patients activate the Hippo signaling pathway by targeting MEF2C, inducing autophagy and ferroptosis in alveolar macrophages (AMs) and exacerbating S-ALI (17). In particular, lncRNA–miRNA interactions, including miRNA sponging, have garnered considerable attention, with multiple ceRNA networks implicated in the initiation and progression of S-ALI. In this review, we systematically summarize the cell type-specific roles of the lncRNA–miRNA–mRNA axis in S-ALI, focusing on alveolar epithelial cells (AECs), pulmonary microvascular endothelial cells (PMVECs), alveolar macrophages (AMs), and neutrophils. Furthermore, we discuss the therapeutic potential of targeting this axis using engineered extracellular vesicles and outline promising directions for future research (**Figure 1**).

## 2 CeRNA networks in S-ALI: cell type-specific roles and therapeutic potential

**Figure 2:**
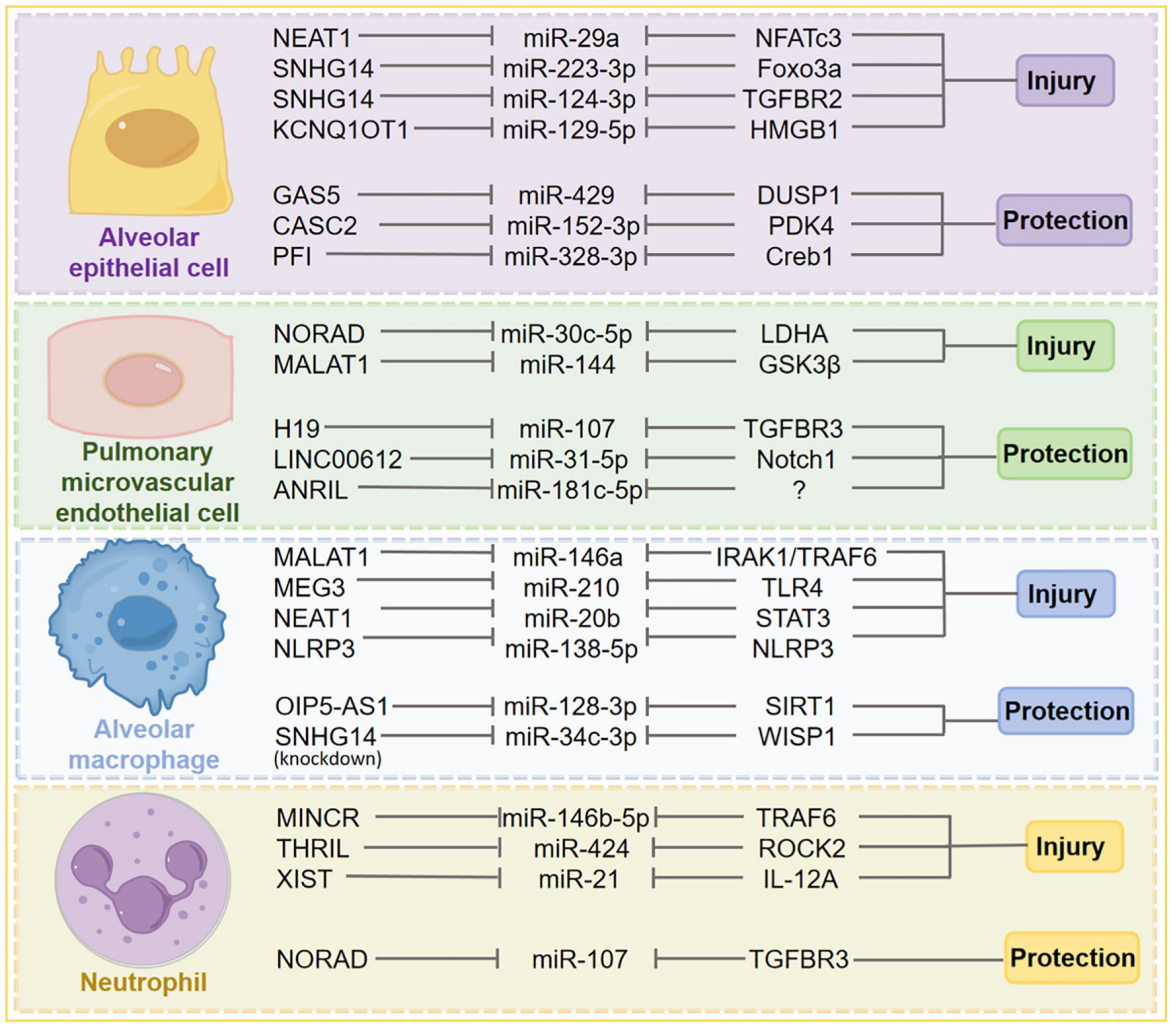
Representative lncRNA–miRNA–mRNA regulatory axes across different cell types in S-ALI. This figure schematically summarizes a selection of representative ceRNA regulatory axes involved in the pathogenesis of S-ALI. It highlights how lncRNAs can sequester specific miRNAs, thereby modulating the expression of target genes (mRNAs) that ultimately drive either cellular injury or confer protection. These intricate interactions are depicted across key pulmonary cell types, including alveolar epithelial cells, pulmonary microvascular endothelial cells, alveolar macrophages, and neutrophils.

The ceRNA network, primarily mediated by lncRNAs acting as molecular sponges for miRNAs, plays a pivotal role in fine-tuning gene expression with significant cell type-specificity in S-ALI. This regulatory axis profoundly influences pathological processes in key pulmonary cells, including AECs, PMVECs, AMs, and neutrophils (**Figure 2**).

### 2.1 Alveolar epithelial cells: apoptosis, inflammation, and barrier dysfunction

AECs form the first barrier of the lung, and their apoptosis and dysfunction are hallmarks of S-ALI, leading to increased alveolar-capillary permeability and pulmonary edema (18, 19). During sepsis, inflammatory damage disrupts both the alveolar epithelium and pulmonary endothelium, resulting in impaired gas exchange (20, 21). Multiple lncRNA-miRNA-mRNA networks regulate these processes through diverse mechanisms. For instance, NEAT1 sponges miR-29a to upregulate NFATc3, promoting inflammation, fibrosis, and apoptosis in AECs (22). Similarly, SNHG14 inhibits miR-223-3p and miR-124-3p, enhancing Foxo3a- and TGFBR2-mediated apoptosis, autophagy, and inflammatory responses (23, 24). Conversely, GAS5 acts as a ceRNA for miR-429 to elevate DUSP1, attenuating MAPK-driven inflammation and apoptosis (25). Notably, lncRNAs such as CASC2 and PFI actively protect against epithelial injury by functioning as ceRNAs for miR-152-3p and miR-328-3p, respectively, thereby preserving alveolar structure and function (26, 27).

Beyond apoptosis and inflammation, the ceRNA network also regulates alveolar epithelial barrier integrity through signaling pathways, autophagy, and cell death mechanisms. For example, SNHG14 and MALAT1 influence autophagic homeostasis and cell cycle progression via miR-223-3p/Foxo3a and miR-129-5p/PAX6 interactions, respectively (24, 28). Additionally, lncRNAs like MIR99AHG and mechanisms involving ferroptosis contribute to barrier remodeling and AECs differentiation (29–31). However, most supporting evidence derives from monocultured cell lines under acute LPS stimulation, highlighting the need for validation in more complex models that incorporate heterotypic cell crosstalk and chronic inflammatory environments. The functional redundancy among lncRNAs targeting shared miRNAs also remains unexplored, presenting both a challenge and opportunity for therapeutic targeting.

### 2.2 Pulmonary microvascular endothelial cells: permeability, apoptosis, and inflammatory signaling

The pulmonary microvascular endothelium is a critical component of the alveolar–capillary barrier, and its dysfunction—marked by increased permeability, leukocyte adhesion, and dysregulated inflammation—is a hallmark of S-ALI (32, 33). Emerging evidence highlights the lncRNA–miRNA–mRNA axis as a central regulator of PMVECs apoptosis, inflammatory signaling, and barrier integrity. For instance, lncRNA NORAD, known for its oncogenic roles, was recently shown to act as a sponge for miR-30c-5p, promoting glycolysis and endothelial dysfunction. Inhibition of NORAD ameliorates LPS-induced injury by modulating lactate dehydrogenase-A (LDHA) (34). Similarly, MALAT1 sponges miR-144, upregulates GSK3β, and drives autophagy, inflammatory mediator release, and PMVECs apoptosis (35). These findings illustrate how lncRNAs sequester miRNAs to exacerbate endothelial injury through diverse molecular pathways.

In ALI, PMVECs apoptosis and inflammatory activation are core pathological events (36, 37). Several lncRNAs act as endogenous protectors by sequestering pathogenic miRNAs. For instance, H19 exerts a strong protective effect by sponging miR-107, which upregulates TGFBR3, reduces apoptosis, suppresses pro-inflammatory cytokines, and promotes anti-inflammatory IL-10, collectively preserving endothelial integrity and attenuating lung injury (38). Pyroptosis also contributes to endothelial injury: the OIP5-AS1/miR-223 axis regulates NLRP3 inflammasome activation, Caspase-1 cleavage, and IL-1β/IL-18 release. Silencing OIP5-AS1 or overexpressing miR-223 inhibits pyroptosis and oxidative stress, improving lung pathology (39). Additional mechanisms include LINC00612/miR-31-5p -mediated activation of Notch signaling and ANRIL/miR-181c-5p-mediated suppression of Bax/Caspase-3 and TNF-α/iNOS pathways, collectively preserving PMVECs function under stress (40, 41).

Despite these advances, key knowledge gaps remain. The temporal dynamics of lncRNA–miRNA interactions across different phases of sepsis—such as the hyper-inflammatory versus immunosuppressive stages—are poorly understood and often overlooked in cross-sectional studies. Furthermore, most evidence derives from rodent models or immortalized cell lines; validation using primary human PMVECs from septic patients is essential to confirm the pathophysiological relevance and therapeutic potential of these regulatory axes.

### 2.3 Alveolar macrophages: polarization, pyroptosis, and immune regulation

AMs serve as pivotal immune sentinels in sepsis, orchestrating the initial inflammatory response (42). Their functional polarization—toward pro-inflammatory (M1) or anti-inflammatory (M2) phenotypes—plays a decisive role in the progression and resolution of lung injury (43, 44). This polarization is intricately modulated by lncRNA–miRNA–mRNA networks, which regulate inflammatory phenotypic switching, pyroptosis, and phagocytic activity.

LncRNAs promote M1 polarization via ceRNA mechanisms, amplifying inflammatory signaling. For instance, MALAT1 sequesters miR-146a, derepressing IRAK1/TRAF6 and activating NF-κB to enhance TNF-α and IL-6 production, exacerbating LPS-induced injury (45). Similarly, MEG3 sponges miR-210 to upregulate TLR4, potentiating NOD-like receptor signaling and IL-1β/IL-6 secretion (46). NEAT1 binds miR-20b, alleviating suppression of STAT3, elevating M1 markers (CD86, iNOS), and suppressing M2 marker CD206 (47). Additionally, lncRNA NONRATT004344, also known as lncRNA NLRP3, sequesters miR-138-5p, thereby activating the NLRP3 inflammasome and and driving caspase-1-mediated pyroptosis via IL-1β/IL-18 maturation (48). These axes—MALAT1/miR-146a/IRAK1, MEG3/miR-210/TLR4, and NEAT1/miR-20b/STAT3 —collectively form a pro-inflammatory network that intensifies lung injury.

Conversely, several lncRNA–miRNA axes play a key protective role by facilitating M2 polarization and promoting inflammation resolution. Silencing MEG3 enhances miR-7b-mediated suppression of NLRP3, reducing caspase-1 activation and IL-1β/IL-18 levels (49). OIP5-AS1 overexpression sponges miR-128-3p, upregulating SIRT1 and inhibiting NF-κB, thereby attenuating TNF-α and IL-6 release and reducing apoptosis (50). SNHG14 knockdown releases miR-34c-3p to inhibit WISP1, lowering IL-1β and TNF-α and ameliorating lung edema (51). Such mechanisms underscore the therapeutic potential of ceRNA networks in restoring immune balance and mitigating excessive inflammation.

Furthermore, lncRNA–miRNA interactions influence AMs phagocytosis and pathogen clearance. For instance, in macrophages, Legionella pneumophila upregulates GAS5, which sponges miR-21 to elevate SOCS6, impairing phagocytosis (52). During influenza infection, lncRNA TCONS_00166432 and circRNA novel_circ_0004733 bind miR-10391, derepressing MAN2A1 and modulating IFN-α/IFN-γ secretion, thereby affecting viral clearance (53). These findings underscore the complex role of ceRNA networks in AMs immune functions. However, the simplistic M1/M2 dichotomy may not capture the full spectrum of macrophage states in sepsis-induced ALI. Future studies using single-cell sequencing are needed to elucidate cell-type-specific ceRNA activities within AMs subpopulations.

### 2.4 Neutrophils: infiltration, NETosis, and apoptotic balance

Neutrophils function as a double-edged sword in S-ALI: while their timely recruitment is essential for host defense, excessive activation contributes to tissue injury through inflammatory infiltration, NETosis, and impaired apoptotic clearance (54–56). These processes are finely regulated by lncRNA–miRNA–mRNA networks that target NF-κB signaling, apoptosis, and key mediators of NET formation (57).

LncRNAs such as MINCR and THRIL enhance neutrophil infiltration by amplifying NF-κB signaling. MINCR sponges miR-146b-5p to upregulate TRAF6, activating the TRAF6/p65 axis and increasing neutrophil infiltration, MPO activity, and pro-inflammatory cytokine release in LPS-induced ALI; these effects are reversed upon MINCR silencing (58). Similarly, THRIL sponges miR-424 to alleviate suppression of ROCK2, activating ROCK2/NF-κB signaling and promoting transendothelial migration and alveolar infiltration. THRIL knockdown reduces neutrophil counts in BALF and mitigates lung injury in septic mice (59). NETosis is modulated by axes such as XIST/miR-21/IL-12A, which elevates IL-12A expression to promote NET formation and inhibit apoptosis, correlating with NET–DNA complexes in clinical samples (60). Bioinformatic analyses suggest that MIR503HG may sponge miR-503 to activate NF-κB/NLRP3 signaling and indirectly upregulate NETosis-related genes like PADI4 and CTSG, though experimental validation is pending (61).

Furthermore, lncRNAs regulate neutrophil apoptosis to balance inflammatory resolution and tissue repair. For instance, H19 is downregulated in septic ALI. Its overexpression sponges miR-107, lifting inhibition on TGFBR3 and activating the SMAD pathway, thereby suppressing neutrophil apoptosis, enhancing IL-10 production, reducing neutrophil accumulation and pro-inflammatory cytokines, and ultimately improving lung histology (38). Despite these insights, targeting neutrophil-related lncRNAs therapeutically remains challenging due to the risk of compromising antimicrobial immunity. Most strategies currently focus on inhibiting infiltration or NETosis, but future efforts should aim for nuanced modulation rather than broad suppression. Additionally, the cellular origins of these lncRNAs—whether neutrophil-intrinsic or acquired via extracellular vesicles—require further investigation.

### 2.5 Extracellular vesicle-mediated ceRNA targeting: therapeutic potential and translational challenges

**Figure 3:**
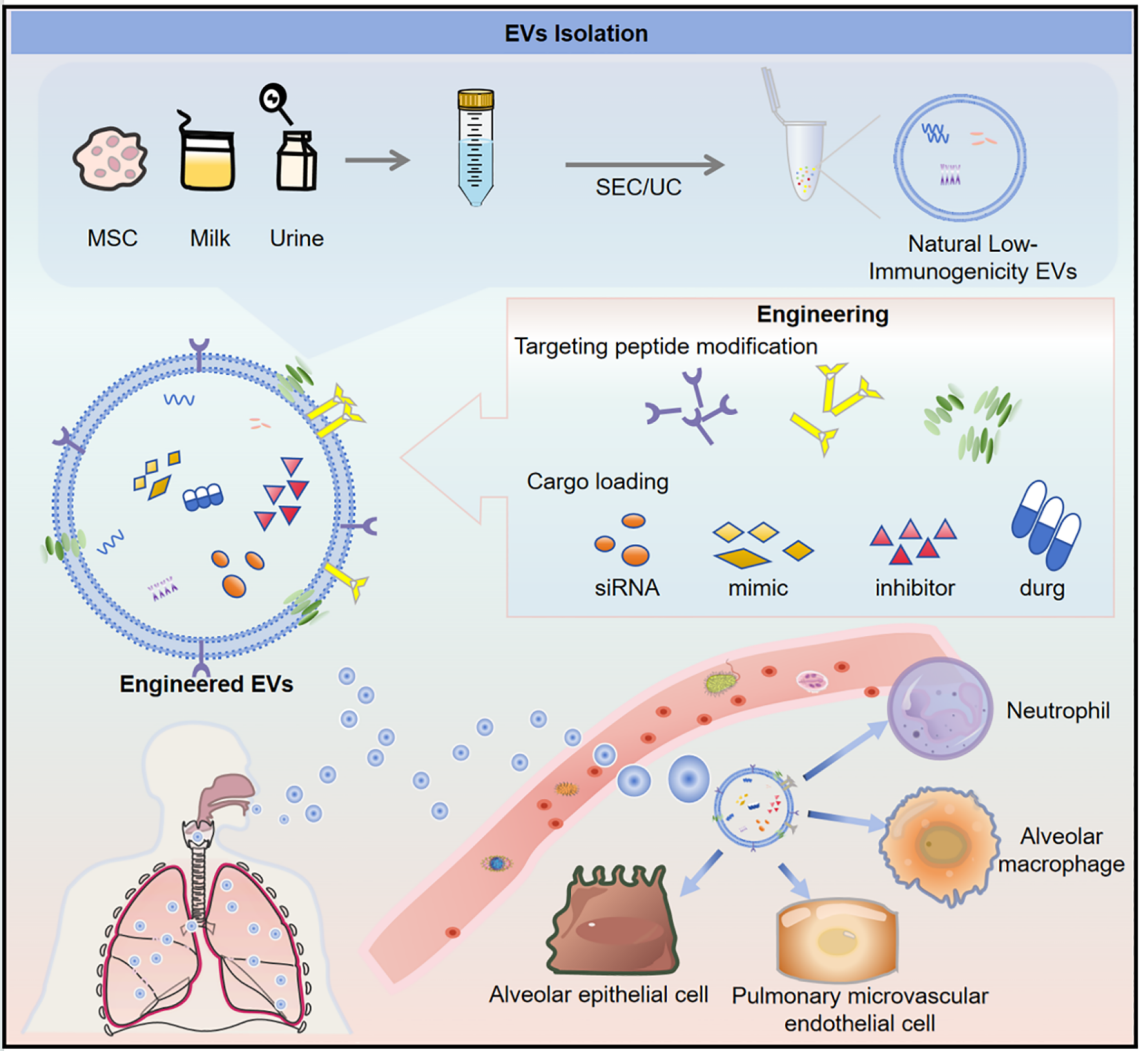
Engineered extracellular vesicles (EVs) for targeted delivery of ceRNA network modulators in the treatment of S-ALI. This figure outlines a strategic process for developing EV-based therapeutics for S-ALI. EVs are first isolated from natural sources with low immunogenicity, such as mesenchymal stem cells (MSCs), milk, or urine, using methods like size exclusion chromatography (SEC) or ultracentrifugation (UC). These natural EVs can then be functionally engineered to enhance their therapeutic potential; this includes modifying their surface with targeting peptides to improve specificity and loading them with various therapeutic cargoes, such as siRNA, miRNA mimics/inhibitors, or drugs. The resulting engineered EVs are designed to precisely deliver their payload to key cellular players in S-ALI—including neutrophils, alveolar macrophages (AMs), alveolar epithelial cells (AECs), and pulmonary microvascular endothelial cells (PMVECs)—thereby offering a targeted treatment strategy.

The intricate cell type-specificity of the ceRNA network in S-ALI underscores the need for precise therapeutic intervention. Extracellular vesicles (EVs), with their excellent biocompatibility and intrinsic targeting capabilities, serve as ideal delivery vehicles for regulators of the lncRNA–miRNA–mRNA axis, offering promising therapeutic strategies for S-ALI (**Figure 3**). As efficient carriers of non-coding RNAs, EVs can modulate target gene expression via the ceRNA mechanism, enabling multi-dimensional interventions (**Table 1**) (62, 63). For instance, natural EVs (e.g., those derived from milk) deliver siRNA or antisense oligonucleotides to silence pro-inflammatory factors such as CCL7, alleviating intestinal inflammation (64). Engineered extracellular vesicles modified with targeting peptides (e.g., alveolar-specific ligands) can inhibit pathogenic miRNAs by delivering anti-miRNA inhibitors. Previously, studies have demonstrated that engineered EVs modified with the lung microvascular endothelial cell-targeting peptide (LET) can efficiently target lung tissues and effectively preserve the endothelial barrier integrity of PMVECs (65). Similarly, conjugating cell-penetrating peptides (CPPs) to the surface of exosomes derived from human hepatocellular carcinoma cells (HepG2) not only enhances the penetration ability of exosomes but also assists in loading antisense oligonucleotides (ASOs) (66). Additionally, EVs can deliver protective RNAs such as miRNA mimics or functional lncRNAs—e.g., miR-146a mimics promoting M2 macrophage polarization to suppress lung injury, or lncRNA DACT3-AS1 enhancing ferroptosis in cancer cells (67, 68). Thus, EV-mediated ceRNA regulation combines natural delivery efficiency with engineering-enhanced targeting, establishing a novel paradigm for molecular therapy.

**Table 1:** Targeted intervention strategies and mechanisms.

Target type	Intervention methods	Core mechanism
lncRNA	Antisense oligonucleotides (ASO)	Blocks lncRNA-target interactions or induces degradation
	siRNA	Degrades lncRNA, reducing its expression level
	CRISPR technology	Knocks out lncRNA genes or represses transcription
	Ribozyme	Catalyzes degradation of lncRNA
	Small molecule inhibitors	Blocks lncRNA-protein/RNA interactions
miRNA	Anti-miRNA oligonucleotides (AMO)	Binds miRNA, blocking its interaction with target mRNA
	miRNA mimics	Restores miRNA function by supplementing expression
	CRISPR technology	Knocks out miRNA genes or regulates transcription
	Small molecule modulators	Regulates miRNA processing (e.g., precursor cleavage) or activity
	RNA sponge	Sequesters miRNA, inhibiting its activity
mRNA	RNA interference (siRNA/shRNA)	Degrades target mRNA and blocks translation
	Antisense oligonucleotides (ASO)	Degrades mRNA or regulates its splicing
	CRISPR-Cas13 system	Cuts and degrades target mRNA
	Ribozyme	Catalyzes degradation of target mRNA
	Antisense oligonucleotides (ASO)	Degrades mRNA or regulates its splicing
	CRISPR-Cas13 system	Cuts and degrades target mRNA
	Ribozyme	Catalyzes degradation of target mRNA
	mRNA translation inhibitors	Blocks ribosome binding or translation elongation

AMO, Anti-miRNA oligonucleotides; ASO, Antisense oligonucleotides; Cas13, CRISPR-associated protein 13; CRISPR, Clustered Regularly Interspaced Short Palindromic Repeats; lncRNA, Long non-coding RNA; miRNA, MicroRNA; mRNA, Messenger RNA; shRNA, Short hairpin RNA; siRNA, Small interfering RNA.

EV-based delivery systems are optimized through targeted modification, activity protection, and innovative sourcing. Surface functionalization with ligands such as mannose enables specific targeting of alveolar macrophages, facilitating miR-23b-3p delivery to inhibit M1 polarization via the Lpar1–NF-κB pathway and alleviate septic ALI (69). Similarly, aptamer- or antibody-conjugated EVs enhance accumulation at disease sites—e.g., MSC-exosomes achieving 66.48% tumor accumulation (70). To ensure nucleic acid stability, milk-derived EVs protect siRNA from degradation, enabling effective gene silencing in intestinal ischemia models (64). Microfluidic technology further improves drug-loading efficiency, reaching up to 90% for doxorubicin (71). Low immunogenicity and homologous targeting make autologous urine exosomes and stem cell-derived exosomes attractive EVs sources (72, 73). These engineering advances address key limitations of conventional delivery systems, such as targeting accuracy and drug-loading stability, thereby facilitating clinical translation.

Co-delivery strategies via EVs enhance therapeutic efficacy through synergistic mechanisms. For example, EVs co-loaded with chemotherapeutic agents (e.g., doxorubicin) and immunomodulatory genes (e.g., IL-12 plasmids) activate antitumor immunity and promote M1 polarization, achieving tumor inhibition rates exceeding 80% (74). Similarly, simultaneous delivery of siRNA and metabolic inhibitors (e.g., 3-bromopyruvate) overcomes drug resistance in KRAS-mutant tumors via an autophagy activation–gene silencing axis (75). Engineered modifications, such as 3D-cultured stem cell EVs enriched with therapeutic proteins like HGF, synergistically enhance alveolar repair through the PI3K–AKT pathway (76). Such approaches reverse resistance mechanisms mediated by factors including FAK, offering a new paradigm for multi-target therapy.

Despite these advances, clinical translation of EV-based ceRNA therapies faces significant challenges. Natural EVs exhibit low accumulation at lesion sites (<5%) and poor penetration across the pulmonary vascular barrier. Inconsistent drug-loading efficiency and low recovery rates in large-scale isolation hinder standardized production. Compensatory activation of target genes (e.g., FAK-mediated resistance) may lead to treatment failure. Moreover, most preclinical studies rely on LPS-induced rodent models, which poorly recapitulate human septic immunopathology and comorbidities. Pharmacokinetic, biodistribution, and safety data in large animal models are scarce. To overcome these barriers, engineering optimizations—such as membrane fusion for high-efficiency mRNA loading, mannose modification for improved macrophage targeting, and microfluidic-based production ensuring batch consistency—are critical. Further research into compensatory mechanisms and off-target effects, alongside robust evaluation in physiologically relevant models, is essential for clinical advancement. Overall, EV-based delivery of ceRNA modulators represents a promising strategy not only to suppress harmful axes but also to enhance endogenous protective networks, offering a dual therapeutic approach for S-ALI.

### 2.6 Experimental models for studying the ceRNA network in sepsis-induced acute lung injury

Understanding the lncRNA-miRNA-mRNA network in S-ALI largely relies on experimental models that recapitulate the key features of human disease. These models are generally categorized into *in vitro* and *in vivo* systems, providing multi-dimensional insights for mechanism exploration and therapeutic development. Among them, EVs, as critical carriers for intercellular communication and disease biomarkers, offer significant supplements and unique advantages to traditional models.

#### 2.6.1 *In vitro* models

*In vitro* studies primarily utilize immortalized cell lines or primary cells, exposing them to stimuli mimicking sepsis, with LPS and TNFα being the most common (77, 78). Models involving alveolar epithelial cells, pulmonary microvascular endothelial cells, macrophages, and neutrophils have played pivotal roles in identifying specific ceRNA interactions. In recent years, EV-mediated research paradigms have emerged as powerful tools for deciphering the intercellular communication functions of ceRNAs. For instance, isolation and analysis of exosomes released by specific cells post-stimulation enable direct identification of their lncRNA and miRNA cargo, thereby linking specific ceRNA axes to intercellular signaling (79, 80). Co-culturing exosomes loaded with or depleted of target non-coding RNAs (ncRNAs) with recipient cells allows direct validation of their transcellular regulatory effects (69, 81). The advantages of these *in vitro* models lie in enabling precision genetic manipulation and high-throughput screening to establish causal relationships within ceRNA axes. However, their notable limitations include oversimplification, failing to fully recapitulate the complexity of the lung microenvironment, dynamic crosstalk between heterogeneous cell populations, and the systemic inflammatory context of sepsis. While *in vitro* models enable precise genetic manipulation and high-throughput screening, their translational relevance is constrained by several methodological limitations. First, most studies rely on immortalized cell lines, which may not reflect the physiological responses of primary human cells. Second, the use of single stimuli (e.g., LPS or TNFα) fails to mimic the polymicrobial and multifactorial nature of clinical sepsis. Third, the lack of heterotypic cell interactions and mechanical forces (e.g., stretch, fluid flow) overlooks critical aspects of alveolar-capillary barrier dysfunction. Future studies should prioritize co-culture systems, organ-on-a-chip platforms, and primary cells from septic patients to enhance physiological relevance while retaining experimental controllability.

#### 2.6.2 *In vivo* models

*In vivo* models, particularly rodent models, are crucial for validating the pathological relevance of ceRNA networks. Intraperitoneal or intratracheal LPS administration is a widely used model of acute injury. The cecal ligation and puncture (CLP) model better mimics the sepsis progression induced by clinical polymicrobial infections (82, 83). These models allow evaluation of ceRNA expression dynamics in the context of whole-body physiology and correlation with lung function, histopathology, and survival outcomes. *In vivo* models also serve as key platforms for investigating the role of endogenous exosomes in S-ALI. Isolation of exosomes from the serum or bronchoalveolar lavage fluid (BALF) of model animals followed by ncRNA omics analysis facilitates the discovery of ceRNA network biomarkers associated with disease stages. Additionally, exosome isolation from the plasma of sepsis patients provides a more clinically relevant sample source for identifying key ceRNA networks linked to disease progression or prognosis (84, 85). Engineered exosomes can be used for targeted *in vivo* delivery of ceRNA regulatory molecules (86). In animal models, the therapeutic efficacy, targeting ability, and safety of engineered exosomes can be validated, representing a critical step bridging basic discoveries and clinical translation (87, 88). This enables researchers not only to observe changes in endogenous ceRNA networks but also to actively intervene and assess their functions. However, exosome-based *in vivo* studies face challenges, including enrichment efficiency at lesion sites, immunogenicity, and consistency in large-scale production, which may impact their clinical predictive value. Nevertheless, translational challenges persist, as immunobiological differences between species and the relative homogeneity of these models cannot fully capture the heterogeneity, comorbidities, and disease stage transitions commonly observed in human sepsis patients.

Despite their utility, current *in vivo* models face significant methodological and translational gaps. The widely used LPS model, while reproducible, primarily reflects hyperinflammatory responses and does not recapitulate the immunosuppressive phase frequently seen in septic patients. The CLP model, though more clinically relevant, exhibits high variability in severity and outcomes, complicating inter-study comparisons. Furthermore, most studies employ young, healthy rodents without comorbidities (e.g., diabetes, aging), limiting their predictive value for the heterogeneous human sepsis population. Standardized reporting of model parameters, inclusion of aged or comorbid animals, and integration of longitudinal multi-omics profiling could improve model fidelity and therapeutic predictability.

Understanding of ceRNA networks in S-ALI has been propelled by reductionist models, which offer mechanistic clarity but are often limited in physiological scope. Overcoming this limitation requires a shift towards integrated methodologies. Future work should leverage patient-derived materials, multi-omics technologies, and sophisticated *in vitro* systems like lung organoids and microfluidic co-cultures. Equally important are commitments to statistical rigor through power analysis, replication in independent cohorts, and comprehensive reporting of all findings. The path forward must also involve designing models that mirror the full spectrum of sepsis, from the initial hyperinflammatory stage to the subsequent immunosuppressive phase, as this clinical alignment is vital for discovering ceRNA pathways amenable to therapeutic intervention.

## 3 Conclusion and perspective

The intricate cell type-specific regulation of the lncRNA-miRNA-mRNA axis in S-ALI, as detailed in this review, underscores its dual role in both driving pathology and providing cellular protection across alveolar epithelial cells, pulmonary microvascular endothelial cells, alveolar macrophages, and neutrophils. This regulatory network fine-tunes critical cellular responses—including inflammation, apoptosis, pyroptosis, and barrier dysfunction— through a complex molecular dialogue centered on miRNA sequestration (**Table 2**). However, a significant limitation of the current evidence is its heavy reliance on monoculture systems and acute LPS challenge models, which inadequately recapitulate the heterotypic cellular crosstalk and dynamic immunopathology of human sepsis. Furthermore, the functional redundancy among multiple lncRNAs targeting the same miRNA (e.g., several lncRNAs regulating miR-223-3p) and the temporal dynamics of these networks across different phases of sepsis remain largely unexplored. Future research employing co-culture models, time-series analyses, and single-cell multi-omics will be essential to delineate the context-specificity and hierarchical importance of these interactions.

**Table 2:** LncRNA-miRNA-mRNA regulatory networks in the pathophysiology of S-ALI.

LncRNA	MiRNA	mRNA	Function	Experiment	Experimental material	Ref.
AECs						
CASC2	miR-152-3p	PDK4	anti-apoptosis, anti-inflammation, anti-oxidative stress	*in vivo* and *in vitro*	Human alveolar epithelial cell line (HPAEpiC); Serum from sepsis-induced ALI patients and healthy controls; Lung tissues from sepsis-induced ALI patients and patients with other lung diseases	(26)
GAS5	miR-429	DUSP1	anti-inflammation, anti-apoptosis	*in vivo* and *in vitro*	Mouse alveolar epithelial cell line (MLE-12); C57BL/6 mice (lung tissues collected)	(25)
KCNQ1OT1	miR-129-5p	HMGB1	pro-inflammation, pro-apoptosis, pro-pulmonary endothelial glycocalyx damage	*in vivo* and *in vitro*	Primary rat type II alveolar epithelial cells (AECII); SD rats (lung tissues and serum collected)	(89)
MALAT1	miR-194-5p	FOXP2	pro-apoptosis	*in vitro*	Human alveolar epithelial cell line (HPAEpiC)	(90)
MALAT1	miR-129-5p	PAX6	pro-apoptosis, pro-inflammation	*in vivo* and *in vitro*	Mouse alveolar epithelial cell line (MLE-12); C57BL/6 mice (lung tissues collected)	(28)
NEAT1	miR-29a	NFATc3	pro-apoptosis, pro-inflammation, pro-fibrosis	*in vitro*	Human alveolar epithelial cell line (HPAEpiC); Human lung adenocarcinoma cell line (A549)	(22)
NEAT1	miR-424-5p	MAPK14	pro-apoptosis, pro-inflammation, pro-oxidative stress	*in vitro*	Human alveolar epithelial cell line (HPAEpiC)	(91)
PFI	miR-328-3p	Creb1	anti-apoptosis	*in vivo* and *in vitro*	Mouse type II alveolar epithelial cell line (MLE-12); Human bronchial epithelial cell line (Beas-2B); C57BL/6 mice (lung tissues collected)	(27)
SNHG14	miR-124-3p	TGFBR2	pro-inflammation, pro-apoptosis	*in vivo*	Human alveolar epithelial cells (A549)	(23)
SNHG14	miR-223-3p	Foxo3a	pro-apoptosis, pro-inflammation, pro-autophagy	*in vivo* and *in vitro*	Human type II alveolar epithelial cell line (A549); LPS-induced C57 mice	(24)
UCA1	miR-182-5p	/	pro-inflammation, pro-apoptosis	*in vivo* and *in vitro*	Human alveolar epithelial cell line (A549); Blood samples from patients undergoing CPB surgery and healthy subjects	(92)
PMVECs						
CHRF	miR-146a	Notch1	pro-inflammation, pro-apoptosis	*in vivo* and *in vitro*	C57BL/6 mice (lung tissues and BALF collected); Mouse pulmonary microvascular endothelial cells (MPVECs)	(93)
LINC00612	miR-31-5p	Notch1	anti-apoptosis, anti-inflammation, anti-oxidative stress	*in vivo* and *in vitro*	Lung tissues from COPD patients, smokers and non-smokers; Human pulmonary microvascular endothelial cells (HPMECs)	(40)
LINC00839	miR-223	NLRP3	pro-pyroptosis, pro-inflammation	*in vivo* and *in vitro*	C57BL/6 mice (lung tissues and BALF collected); Mouse pulmonary microvascular endothelial cells (MPVECs)	(94)
NORAD	miR-30c-5p	LDHA	pro-apoptosis	*in vivo* and *in vitro*	Human pulmonary microvascular endothelial cells (HPMECs); A549 cells; Pulmonary microvascular endothelial cells from ALI patients and healthy subjects; SD rats (lung tissues and pulmonary microvascular endothelial cells collected)	(34)
ANRIL	miR-181c-5p	No specified	anti-apoptosis, anti-inflammation, anti-vasoactive factor release	*in vitro*	Mouse pulmonary microvascular endothelial cells (mPMVECs)	(41)
H19	miR-107	TGFBR3	anti-apoptosis, anti-inflammatory factor release	*in vivo* and *in vitro*	C57BL/6 mice (lung tissues and BALF collected); Mouse pulmonary microvascular endothelial cells (PMVECs)	(38)
MALAT1	miR-144	GSK3β	pro-apoptosis, pro-autophagy, pro-inflammatory factor release	*in vivo* and *in vitro*	C57BL/6 mice (lung I/R model, pretreated with propofol); Primary mouse pulmonary microvascular endothelial cells (PMVECs, H/R model, pretreated with propofol)	(35)
MEG3	miR-421	DFFB	pro-apoptosis	*in vitro*	Human pulmonary microvascular endothelial cells (HPMECs); Human bronchial epithelial cells (HBECs)	(95)
MIR3142HG	miR-450b-5p	HMGB1	pro-apoptosis, pro-inflammation	*in vitro*	Human pulmonary microvascular endothelial cells (HPMECs)	(96)
OIP5-AS1	miR-223	NLRP3	anti-proliferation, pro-apoptosis, pro-pyroptosis, pro-inflammation, pro-oxidative stress	*in vivo* and *in vitro*	Serum from ALI/ARDS patients and healthy subjects; Human pulmonary microvascular endothelial cells (HPMECs); SD rats (lung tissues collected)	(39)
PICALM-AU1	miR-144-3p	ZEB1	pro-EndMT, pro-proliferation, pro-migration, pro-barrier dysfunction, pro-adhesion molecule regulation	*in vivo* and *in vitro*	SD rats (HPS model); Serum from human HPS patients and patients with chronic liver disease; Rat pulmonary microvascular endothelial cells (PMVECs); Mouse intrahepatic biliary epithelial cells (MIBECs)	(97)
SNHG16	miR-372-3p/miR-373-3p	MTCH2	pro-proliferation, anti-apoptosis, anti-vascular endothelial permeability	*in vivo* and *in vitro*	Mouse lung I/R model; Human pulmonary microvascular endothelial cells (HPMECs), type II alveolar epithelial cells (AECs, OGD/R model)	(98)
SNHG3	miR-186-5p	Wnt	pro-apoptosis, pro-barrier permeability	*in vitro*	Human pulmonary microvascular endothelial cells (HPMVECs, sepsis-related ALI model)	(99)
TUG1	miR-34b-5p	GAB1	anti-apoptosis, anti-inflammation	*in vivo* and *in vitro*	C57BL/6 mice (lung tissues collected); Mouse pulmonary microvascular endothelial cells (PMVECs); Serum from ARDS patients and healthy subjects	(100)
TUG1	miR-9a-5p	BCL2L11	pro-apoptosis	*in vitro*	Human pulmonary microvascular endothelial cells (HPMECs)	(101)
AMs						
GAS5	miR-29c	HMGB1	pro-M1 polarization, pro-inflammatory phenotype	*in vivo* and *in vitro*	Mouse alveolar macrophage cell line (MH-S); Primary mouse alveolar macrophages (AMs, isolated from BALF); C57BL/6 mice	(102)
GAS5	miR-21	SOCS6	pro-phagocytosis, anti-chemotaxis	*in vitro*	Mouse macrophage cell line (RAW264.7, stimulated with MIP from Legionella pneumophila)	(52)
H19	miR-423-5p	FOXA1	pro-M1 polarization, pro-inflammatory phenotype	*in vivo* and *in vitro*	Male Wistar rats (divided into 4 groups); Mouse alveolar macrophage cell line (MH-S); Mouse alveolar epithelial cell line (MLE-12)	(103)
MALAT1	miR-146a	IRAK1/TRAF6	pro-M1 polarization, pro-inflammatory phenotype	*in vivo* and *in vitro*	Mouse alveolar macrophage cell line (MH-S); Mouse alveolar epithelial cell line (MLE-12); ALI rat model (lung tissues and BALF collected)	(45)
MEG3	miR-210	TLR4	pro-M1 polarization, pro-inflammatory phenotype	*in vitro*	Porcine alveolar macrophage cell line (PAMs, 3D4/21)	(46)
MEG3	miR-7b	NLRP3	pro-inflammatory phenotype	*in vivo* and *in vitro*	Mouse alveolar macrophage cell line (NR8383); Male C57BL/6J mice (lung tissues and BALF collected)	(49)
MEG3 - 4	miR-138	IL - 1β	pro-inflammatory response (early), anti-inflammatory response (late, negative feedback)	*in vivo* and *in vitro*	Alveolar macrophages, lung epithelial cells (cultured *in vitro*); Mice (pulmonary bacterial infection model)	(104)
NEAT1	miR-20b	STAT3	anti-M1 to M2 transition, pro-inflammatory phenotype	*in vivo* and *in vitro*	Mouse alveolar macrophage cell line; C57BL/6 mice (VILI model)	(47)
NLRP3	miR-138-5p	NLRP3	pro-inflammatory phenotype	*in vivo* and *in vitro*	Rat alveolar macrophage cell line (NR8383); Male SD rats (lung tissues and BALF collected)	(48)
OIP5-AS1	miR-128-3p	SIRT1	anti-apoptosis, anti-inflammation	*in vivo* and *in vitro*	Rat alveolar macrophage cell line (NR8383); Rat type II alveolar cell line (RLE-6TN); SD rats (sepsis model, lung tissues and BALF collected)	(50)
TCONS_00166432	miR-10391	MAN2A1	pro-M1 polarization, pro-inflammatory phenotype	*in vitro*	Porcine alveolar macrophage cell line (3D4/21)	(53)
Neutrophil						
H19	miR-107	TGFBR3	anti-neutrophil infiltration, anti-inflammation	*in vivo*	Mice (sepsis-induced acute lung injury model, lung tissues and BALF collected)	(38)
MINCR	miR-146b-5p	TRAF6	pro-neutrophil activation, pro-infiltration, pro-toxicity (NETs formation)	*in vivo* and *in vitro*	Mice (lung tissues and BALF collected); Human small airway epithelial cells (SAECs)	(58)
THRIL	miR-424	ROCK2	pro-neutrophil migration, pro-inflammatory infiltration	*in vivo* and *in vitro*	Mouse pulmonary microvascular endothelial cells (MPVECs, stimulated with LPS); CLP septic mice (lung tissues and BALF collected)	(59)
XIST	miR-21	IL-12A	pro-neutrophil activation, pro-NETs formation, anti-apoptosis	*in vivo* and *in vitro*	BALF and BAL cells from PGD and non-PGD patients; SD rats (left lung transplantation model); Peripheral blood neutrophils (PMNs) from healthy volunteers	(60)

AECs, Alveolar Epithelial Cells; ALI, Acute Lung Injury; AMs, Alveolar Macrophage; ARDS, Acute Respiratory Distress Syndrome; ASO, Antisense oligonucleotides; BALF, Bronchoalveolar Lavage Fluid; CLP, Cecal Ligation and Puncture; CPB, Cardiopulmonary Bypass; COPD, Chronic Obstructive Pulmonary Disease; CRISPR, Clustered Regularly Interspaced Short Palindromic Repeats; EndMT, Endothelial-to-Mesenchymal Transition; HPS, Hepatopulmonary Syndrome; H/R, Hypoxia/Reoxygenation; I/R, Ischemia/Reperfusion; LncRNA, Long non-coding RNA; LPS, Lipopolysaccharide; MiRNA, MicroRNA; mRNA, Messenger RNA; NETs, Neutrophil Extracellular Traps; OGD/R, Oxygen-Glucose Deprivation/Reoxygenation; PGD, Primary Graft Dysfunction; PMNs, Polymorphonuclear Neutrophils; PMVECs, Pulmonary Microvascular Endothelial Cell; SD, Sprague-Dawley; shRNA, Short hairpin RNA; siRNA, Small interfering RNA.

The therapeutic potential of targeting the ceRNA network using engineered extracellular vesicles represents a promising frontier for S-ALI treatment. EVs offer a natural delivery platform with superior biocompatibility and the potential for cell-specific targeting through surface modifications. Preclinical studies have demonstrated the feasibility of using EV-encapsulated miRNA mimics, inhibitors, or lncRNAs to modulate pathogenic axes in animal models. For instance, delivering miR-23b-3p via mannose-modified EVs to alveolar macrophages can suppress pro-inflammatory polarization. However, major translational challenges persist, including low natural accumulation rates at lesion sites (<5%), batch-to-batch variability in EV production, and potential off-target effects. Moreover, the compensatory activation of alternative pathways (e.g., FAK-mediated resistance) may undermine monotherapeutic strategies. Advancing EV-based therapeutics will require innovative engineering approaches—such as microfluidic-based loading, biomimetic membrane modifications, and co-delivery systems—to enhance targeting precision, payload stability, and synergistic efficacy.

In addition, a question worthy of in-depth exploration is whether the ceRNA network dynamically evolves across different clinical phases of sepsis, such as the hyperinflammatory phase and the immunosuppressive phase. Most current studies are based on acute stimulation models, such as LPS, which cannot fully reflect the persistent immune and metabolic remodeling during sepsis. For example, certain pro-inflammatory lncRNAs, such as NEAT1 and MALAT1, may be significantly upregulated in the early hyperinflammatory phase, but may be suppressed or undergo functional switching in the late immunoparalysis phase. Similarly, miRNA expression profiles may change with inflammatory status, organ function grades, and therapeutic responses. Future research should integrate time-series clinical samples (plasma and BALF from patients with different SOFA scores or infection stages) with single-cell sequencing technology to systematically dissect the phase-specific regulatory patterns of the ceRNA network, thereby providing a basis for stage-targeted therapy.

Beyond the mechanistic and therapeutic considerations, the pathophysiological relevance of identified ceRNA networks warrants critical appraisal. Many proposed interactions are predicated on luciferase reporter assays and bioinformatic predictions without rigorous *in vivo* validation. The transition from murine models to human sepsis is particularly problematic, as murine immunobiology and the homogeneity of LPS models poorly mirror the heterogeneity and comorbidities of human patients. Additionally, the origin of dysregulated lncRNAs in specific cell types—whether intrinsically expressed or externally acquired via EV-mediated transfer—is often unclear. Future studies should prioritize the use of primary human cells, sophisticated sepsis models (e.g., CLP with secondary infection), and spatial transcriptomics to validate these networks in a more clinically relevant context. Assessing the correlation between specific ceRNA axis components and clinical outcomes in septic patients could further strengthen their therapeutic candidacy. Furthermore, advancing EV-based ceRNA therapies into the clinic will require addressing key translational challenges beyond biological mechanisms. Regulatory frameworks for EV-based products remain under development, necessitating clear guidelines for characterization, potency, and quality control. Safety assessments must rigorously evaluate off-target effects, immunogenicity, and long-term biodistribution. Manufacturing scalability and batch-to-batch consistency are critical for clinical-grade production. Encouragingly, early-phase clinical trials exploring EV-based therapies for inflammatory diseases are underway, which may pave the way for future trials targeting ceRNA networks in S-ALI.

Looking forward, a holistic understanding of the ceRNA network’s role in sepsis must extend beyond the lung. Given the systemic nature of sepsis, similar regulatory axes likely operate in other organs affected by sepsis-induced dysfunction, such as the kidney, liver, and heart. Exploring common pathogenic pathways could reveal pan-organ therapeutic targets. Moreover, integrating artificial intelligence with multi-omics data may help predict context-specific ceRNA interactions and optimize EV design for personalized delivery. Addressing these challenges will require interdisciplinary collaboration among molecular biologists, bioengineers, and clinical intensivists to translate these compelling mechanistic insights into effective therapies for S-ALI.

In summary, the lncRNA-miRNA-mRNA regulatory axis is a critical determinant of cell type-specific responses in S-ALI, influencing a spectrum of pathological processes from inflammation and cell death to immune dysregulation. While preclinical studies highlight the therapeutic potential of targeting this network, particularly via engineered extracellular vesicles, significant knowledge gaps and translational hurdles remain. Overcoming these challenges will necessitate the validation of ceRNA interactions in more physiological models, the development of sophisticated EV-based delivery systems, and a broader exploration of these mechanisms across sepsis-related organ injuries. Ultimately, harnessing the full potential of this intricate regulatory network may pave the way for precision therapies aimed at improving the dismal outcomes of sepsis-associated lung injury.
